# Religious Factors and Hippocampal Atrophy in Late Life

**DOI:** 10.1371/journal.pone.0017006

**Published:** 2011-03-30

**Authors:** Amy D. Owen, R. David Hayward, Harold G. Koenig, David C. Steffens, Martha E. Payne

**Affiliations:** 1 Center for the Study of Aging and Human Development, Duke University Medical Center, Durham, North Carolina, United States of America; 2 Department of Psychiatry and Behavioral Sciences, Duke University Medical Center, Durham, North Carolina, United States of America; 3 Neuropsychiatric Imaging Research Laboratory, Duke University Medical Center, Durham, North Carolina, United States of America; 4 Department of Medicine, Duke University Medical Center, Durham, North Carolina, United States of America; National Institute on Aging Intramural Research Program, United States of America

## Abstract

Despite a growing interest in the ways spiritual beliefs and practices are reflected in brain activity, there have been relatively few studies using neuroimaging data to assess potential relationships between religious factors and structural neuroanatomy. This study examined prospective relationships between religious factors and hippocampal volume change using high-resolution MRI data of a sample of 268 older adults. Religious factors assessed included life-changing religious experiences, spiritual practices, and religious group membership. Hippocampal volumes were analyzed using the GRID program, which is based on a manual point-counting method and allows for semi-automated determination of region of interest volumes. Significantly greater hippocampal atrophy was observed for participants reporting a life-changing religious experience. Significantly greater hippocampal atrophy was also observed from baseline to final assessment among born-again Protestants, Catholics, and those with no religious affiliation, compared with Protestants not identifying as born-again. These associations were not explained by psychosocial or demographic factors, or baseline cerebral volume. Hippocampal volume has been linked to clinical outcomes, such as depression, dementia, and Alzheimer's Disease. The findings of this study indicate that hippocampal atrophy in late life may be uniquely influenced by certain types of religious factors.

## Introduction

Religion is considered an important part of life for many Americans, with 92% reporting a belief in God or a universal spirit, 83% belonging to a religious group, and 59% reporting that they pray at least daily [1]. Research on the neurological processes involved in spiritual beliefs and practices has been growing, but studies examining possible religious or spiritual correlates of structural neuroanatomy have been rare. Specific changes in brain function have been associated with practices including meditation [2], [3], [4], [5], [6], prayer [6], [7], and a variety of religious and spiritual experiences [8], [9], [10], [11]. Several brain regions, including the hippocampus [4], have also been implicated in religious experiences and practice [4], [5], [9], [12], [13], [14], [15], [16]. A small number of studies have found that religious beliefs, practices, and experiences are correlated with the volume of specific brain regions, but the focus has been limited to hyper-religiosity in temporal lobe epilepsy patients [17], [18] and beliefs about the nature of God [19]. The current study extends this research by examining relationships between a broad range of religious factors and hippocampal volumes, including religious group membership, religious practices, and life-changing religious experiences in a sample of older adults.

The hippocampus has several important functions, including spatial, contextual, and episodic learning and memory [20], [21], [22], [23], [24], [25], [26], [27]. The hippocampus may also influence the generation of attention and emotion through connections with the amygdala [28], and moderate cortical arousal and responsiveness through interconnections with the amygdala, hypothalamus, prefrontal cortex, and other areas [28]. Global cerebral atrophy occurs as a result of aging [29], but atrophy rates differ between brain regions [30], [31]. Rates of atrophy for the hippocampus have been found to accelerate during late life [29]. Research indicates that hippocampal volumes may be affected by exposure to elevated glucocorticoids, particularly cortisol, a hormone released in response to stress [32], [33], [34], [35], [36], [37], and that cumulative cortisol exposure may lead to hippocampal atrophy through various pathways [33], [34], [35], [36]. This atrophy has been associated with mental health outcomes, including depression [38], [39], [40], [41], [42], [43] and dementia [44], [45], [46], [47], [48], [49] in later life. Studies have also identified the hippocampus as a brain region potentially involved in religious beliefs and spiritual practices. Initial findings indicate that the hippocampus is activated during meditation [4], and that larger hippocampal volumes are associated with long-term meditation practice [28], [50]. Among certain epilepsy patients, smaller hippocampal volumes have also been associated with hyper-religiosity [18].

Building on evidence from research with meditation and temporal lobe epilepsy, within the context of hypothesized mechanisms of stress and glucocorticoids, this study focused on the potential role of religious factors in hippocampal atrophy. The objective of the present study was to delineate the pattern of prospective relationships between religious factors and hippocampal volume change in a large sample of older adults.

## Methods

### Ethics statement

The Psychiatry Institutional Review Board of Duke University Medical Center has approved this research. After complete description of the study to the subjects, informed written consent was obtained. All clinical investigation has been conducted according to the principles expressed in the Declaration of Helsinki.

### Participants

Participants were 268 men and women aged 58 and over, recruited for the NeuroCognitive Outcomes of Depression in the Elderly (NCODE) study. Details of recruitment for this ongoing longitudinal study are described elsewhere [38]. Participants included two groups, those meeting DSM-IV [51] criteria for major depressive disorder and never-depressed comparison participants. Exclusion criteria included concurrent diagnosis of other psychiatric or neurological illness, significant cognitive impairment, and substance abuse. Requirements for inclusion in the non-depressed group were no evidence of a diagnosis of depression or self-report of neurological or depressive illness. Participants included in these analyses were enrolled between November 1994 and January 2005, and provided two or more sets of MRI measurements.

MRI scans were acquired every two years, and religious, psychosocial, and demographic data were collected at baseline and annually, using a structured psychiatric interview. Length of time between baseline and final available MRI measurement ranged from 2–8 years (mean 4.19).

### Religion measures

Religious factors assessed at baseline included (1) frequency of public worship, (2) frequency of private religious activity (prayer, meditation, or Bible study), (3) religious group membership. Religious factors assessed at baseline and annually included (4) born-again status and (5) life-changing religious experiences. Born-again status was assessed with the question, “Are you a born-again Christian?” This was defined as: “A conversion experience, i.e., a specific occasion when you dedicated your life to Jesus.” Participants responding no were assessed for life-changing religious experiences with the question, “Have you ever had any other religious experience that changed your life?” Participants' responses changed over time; thus were categorized as: 1) no born again status or life-changing religious experience, 2) baseline born-again status, 3) new born-again status (i.e., responded no to born-again question at baseline, but yes at a later interview), 4) baseline life-changing religious experience, and 5) new life-changing religious experience. Religious group membership was classified as Catholic, Protestant, Other, or None. Because of the high degree of overlap between Protestant group membership and born-again status, the Protestant group was further divided into born-again and non born-again subcategories.

### Image acquisition and analysis

All subjects were imaged with a 1.5-T, whole body MRI system (Signa; GE Medical Systems, Milwaukee, WI) using the standard head (volumetric) radiofrequency coil. Two sets of dual-echo, fast spin-echo acquisitions were obtained: one in the axial plane for morphometry of cerebrum and another in a coronal oblique plane for measurement of the hippocampus. Imaging acquisition parameters [52], volumetry of hippocampus and cerebrum [53], and the GRID software program used in analysis [54] have been described previously. Image analysis was performed at the Duke Neuropsychiatric Imaging Research Laboratory. Total cerebral volume was defined as white matter, gray matter, and cerebrospinal fluid in both cerebral hemispheres.

### Covariates

Psychosocial and demographic covariates were included in these analyses, as well as baseline total cerebral volumes as a proxy for head size. Psychosocial factors assessed included stress (global self-reported stress experienced over the past 6 months), social support (a composite variable, primarily level of satisfaction with personal relationships [55], [56]), and depression status (membership in depressed or non-depressed group). Demographic factors assessed included age, sex, self-reported race (dichotomized as white and non-white), years of education, and duration in the study.

### Data analysis

Multiple linear regression analyses were conducted to assess relationships between religious variables and hippocampal volume change between baseline and final MRI measurement, controlling for psychosocial and demographic covariates, and baseline total cerebral volume. Left and right hippocampal volumes were calculated separately; volume change measures were computed by subtracting baseline region volume from final region volume.

## Results

Descriptive statistics for the study sample are presented in Table 1 (N = 268), including demographics, religious factors, covariates, and brain volumes. Table 2 presents longitudinal regression models of religious factors and covariates on change in left and right hippocampal volumes. Positive model coefficients indicate less atrophy over time. Reported life-changing religious experience at baseline was associated with greater atrophy between baseline and follow-up in the left and right hippocampus (left: *b* = −0.45, *P*<.001; right: *b* = −0.32, *P* = .012). Born-again Protestant group membership at baseline was associated with greater atrophy in the left and right hippocampus compared with non born-again Protestant group membership (left: *b* = −0.15, *P* = .046; right: *b* = −0.15, *P* = .050). Catholic group membership (n = 22) (*b* = −0.22, *P* = .046) and no religious group membership at baseline (n = 19) (*b* = −0.28, *P* = .046) were also associated with greater atrophy in the left hippocampus over time compared with non born-again Protestant group membership.

**Table 1:** Descriptive statistics (N = 268).

	N (%) or Mean (SD)
Brain Volume	
Left hippocampus baseline (mL)	2.96 (0.43)
Left hippocampus final (mL)	2.97 (0.48)
Right hippocampus baseline (mL)	3.11 (0.43)
Right hippocampus final (mL)	3.06 (0.52)
Total cerebrum baseline (mL)	1151.66 (124.75)
Gender	
Female	182 (67.9%)
Male	86 (32.1%)
Age (years)	69.21 (6.44)
Race	
Asian	3 (1.1%)
Black	20 (7.5%)
Native American	1 (0.4%)
White	234 (87.3%)
Other	10 (3.7%)
Education (years)	14.64 (2.49)
Time in study (years)	4.49 (1.89)
Stress	4.88 (2.58)
Social Support	24.97 (3.51)
Religion	
Private practice	2.88 (1.89)
Public worship	2.97 (1.76)
Affiliation	
Non born-again Protestant	113 (42.2%)
Born-again Protestant	97 (36.2%)
Catholic	22 (8.2%)
Other religion	17 (6.3%)
No religion	19 (7.1%)
Religious experience	
Born-again (baseline)	97 (36.2%)
LCRE^a^ (baseline)	13 (4.9%)
Born-again (new)	22 (8.2%)
LCRE^a^ (new)	23 (8.6%)

a Life-changing Religious Experience.

**Table 2:** Regression Analyses of Religious Factors and Changes in Hippocampal Volume (N = 268).

	Left Hippocampus			Right Hippocampus		
	*b*	(SE)	β	*b*	(SE)	β
Intercept	0.45	(0.53)		0.48	(0.53)	
Religion/Spirituality						
Born-again^a^ (new)	−0.05	(0.12)	0.03	−0.21	(0.12)	−0.12
LCRE^b^ (baseline)	−0.45***	(0.12)	−0.22	−0.32	(0.13)	−0.16
LCRE^b^ (new)	−0.01	(0.12)	−0.01	−0.15	(0.12)	−0.08
Born-again^a^ (baseline)	−0.15*	(0.08)	−0.16	−0.15*	(0.08)	−0.16
Catholic	−0.22*	(0.11)	−0.13	−0.12	(0.11)	−0.07
Other	0.06	(0.12)	0.04	−0.05	(0.12)	−0.03
None	−0.28*	(0.12)	−0.13	−0.20	(0.12)	−0.10
Private practice	0.02	(0.02)	0.06	0.03	(0.02)	0.11
Public worship	−0.002	(0.02)	0.01	0.001	(0.02)	0.001
Covariates						
Depression status	−0.09	(0.09)	0.09	−0.08	(0.09)	0.08
Social support	0.01	(0.01)	0.09	0.01	(0.01)	0.09
Stress	0.01	(0.01)	0.03	0.003	(0.01)	0.02
Total brain size	0.0001	(0.001)	0.03	0.001	(0.001)	0.004
Age	−0.01*	(0.004)	−0.16	−0.01	(0.004)	−0.18
Duration in study	0.001	(0.02)	0.01	−0.01	(0.02)	0.02
Sex (female)	0.10	(0.08)	0.10	0.04	(0.08)	0.04
Race (White)	−0.004	(0.08)	−0.01	0.04	(0.08)	0.03
Education	−0.001	(0.01)	−0.01	0.004	(0.01)	0.02

*p<.05, ** *p*<.01,

***p<.001.

a Born-again labels refer to Protestants reporting born-again status.

b Life-changing Religious Experience.

## Discussion

The findings of this study indicate that certain religious factors may influence longitudinal change in hippocampal volume during late life. Greater hippocampal atrophy over time was predicted by baseline identification as born-again Protestants, Catholics, or no religious affiliation, compared with Protestants who were not born-again. Greater hippocampal atrophy was also predicted by reports at baseline of having had life-changing religious experiences. These longitudinal associations were not explained by baseline psychosocial or psychiatric factors (social support, stress, and depression status), demographic factors, duration in the study, or total baseline cerebral volume. Frequency of public and private religious activity did not predict changes in hippocampal volume.

One way of interpreting these findings is within the context of the hypothesized impact of cumulative stress on the hippocampus. While some religious variables have been found to be associated with positive mental health [57], [58], [59], other religious factors may be a source of stress [19], [60], [61], [62], [63], [64]. Research on biological pathways by which stress may influence hippocampal volumes has primarily explored neuronal death [32], [65], [66], [67], [68], [69], decreased neurogenesis [70], [71], [72], [73] and dendritic retraction [74], [75]. The glucocorticoid vulnerability hypothesis proposes that chronic stress alters the hippocampus by elevating levels of glucocorticoids, which in turn extends the time period during which the hippocampus is susceptible to damage from various sources [37]. The measure of stress used in this study was not correlated with changes in hippocampal volume, possibly due to the fact that it captured acute rather than cumulative stressors. Research indicates that relationships between stress and hippocampal volume likely operate at the level of cumulative rather than acute stress, leaving the cumulative stress framework a plausible interpretation of these results.

Greater hippocampal atrophy was observed longitudinally in this study among born-again Protestants, Catholics, and those reporting no religious affiliation, compared with non born-again Protestants. These findings may reflect potential cumulative stress associated with being a member of a religious minority. Though religious factors have been associated with positive mental health [59], [76], [77], studies have shown members of religious minority groups may also experience stressors related to these group affiliations [78], [79], [80]. Greater hippocampal atrophy was also found to be longitudinally associated with reported life-changing religious experiences. Spiritual experiences not easily interpreted within an existing cognitive framework or set of religious beliefs have been shown in previous research to be detrimental to subjective well-being [81]. Such experiences have the capacity to produce doubts regarding previously unquestioned convictions, potentially inducing cumulative stress even if the experience was subjectively positive. If the experience prompts a change in religious groups, existing social networks may also be disrupted. Thus, as possible sources of cumulative stress, both minority religious group membership and life-changing religious experiences may contribute to conditions that are deleterious for hippocampal volume.

These findings can be interpreted within the framework of previous studies identifying the hippocampus as a brain region potentially involved in religious or spiritual beliefs and practices. Using PET and MRI data, studies of meditation indicate that the hippocampus has been found to be activated during meditative states, compared to control states [4], [16]. Structurally, among meditation practitioners (compared to non-practitioner controls), significantly larger volumes [28], [50] and higher gray matter concentrations [28] have been found in regions activated during meditation, including the right hippocampus. The current study did not find an association between change in hippocampal volume and frequency of spiritual activities, possibly reflecting the potential of varying spiritual practices to affect neuroanatomy differently. Research on temporal lobe epilepsy indicates that features of hyper-religiosity may be positively associated with hippocampal atrophy, but findings are mixed [17], [18]. Associations found in the current study between life-changing religious experiences (but not frequency of religious practices) and hippocampal atrophy are consistent with a previous finding that the content and intensity of religious experiences (but not frequency of religious activities), differed between regular churchgoers and temporal-lobe epilepsy patients with hyper-religious features [82], symptoms linked to hippocampal atrophy in some studies [18].

The relatively large sample size, longitudinal design, and the assessment of a range of religious and psychosocial factors are strengths of this study. Limitations include the geographically and religiously constrained nature of the sample (largely Southeastern Protestant Christians), as well as the small sample size of participants reporting a life-changing religious experience. The image acquisition used in this study is also limited to the technology available when it began in 1994, which was retained throughout the study in order to have comparable scans for longitudinal analyses. Future research on qualitative aspects of life-changing religious experiences could provide critical insight into the particular features of religion underlying the observed relationships with hippocampal volume. In addition, comprehensive cognitive testing in future studies could help determine the role of cognitive performance in both late life religious experiences and hippocampal volume.

This study is among the first to examine religious and spiritual correlates of structural neuroanatomy, identifying several understudied factors associated with hippocampal atrophy. Religious factors, including religious group membership and life-changing religious experiences, but not frequency of public and private religious practices, were longitudinally associated with hippocampal atrophy. Atrophy in this region has important clinical implications, having been identified as a marker of late life mental health problems such as depression [38], [39], [40], [41], [42], [43] and dementia [44], [45], [46], [47], [48], [49]. These results may reflect an impact of cumulative stress on hippocampal volume. Mechanisms for these results, such as the elucidation of potential glucocorticoid stress pathways leading to atrophy, need to be more clearly identified, making the interpretation of these findings necessarily speculative. Future research exploring neuroanatomical changes in late life should not overlook the potential impact of religious factors, which remain relevant for a substantial proportion of the US population.
